# Solvent Extraction of Metals in the Circular Economy: Enhancing Resource Efficiency and Sustainability

**DOI:** 10.1155/tswj/8446347

**Published:** 2026-07-03

**Authors:** Anup Basnet Chetry

**Affiliations:** ^1^ Graduate School of Science and Technology, Mid-West University, Surkhet, Nepal

## Abstract

The integration of solvent extraction techniques into the circular economy framework offers a sustainable approach to addressing resource efficiency and metal recovery challenges. This review explores the role of solvent extraction in minimizing environmental impacts while maximizing the recovery of critical metals from waste streams, including electronic waste, industrial residues, and end‐of‐life products. Emphasis is placed on recent advancements in solvent systems, such as biodegradable and smart solvents, and their potential to enhance process efficiency and sustainability. The paper also highlights hybrid extraction systems and advanced separation techniques that complement solvent extraction, enabling higher recovery rates and improved selectivity. Furthermore, the significance of government initiatives, industry collaborations, and investment in sustainable technologies is discussed, underscoring their role in fostering innovation and driving widespread adoption of solvent extraction processes. By integrating solvent extraction with circular economy principles, this review advocates for a holistic approach to sustainable resource management and the development of resilient supply chains, contributing to long‐term economic and environmental sustainability.

## 1. Introduction

The global drive toward sustainable industrial practices has brought the concept of the circular economy to the forefront of environmental and economic strategies [1, 2]. The circular economy is aimed at minimizing waste [3], optimizing resource efficiency [4], and promoting the reuse and recycling of materials [5], thereby offering a viable solution to the challenges posed by resource depletion and environmental degradation [6]. One critical technology supporting this transition is solvent extraction (SX), a well‐established method for the recovery of metals from both primary and secondary sources, such as industrial waste and electronic waste (e‐waste). [7, 8] The circular economy has emerged as a transformative model for rethinking industrial processes, resource consumption, and waste management [9]. By prioritizing reuse, recycling, and the efficient use of materials, the circular economy provides a framework that minimizes environmental impact while maintaining economic growth. [10] A key enabler of this model is the application of SX—a widely used hydrometallurgical technique that allows for the selective separation and recovery of valuable metals from various sources [11, 12]. SX plays a pivotal role in achieving the goals of the circular economy by enabling the selective recovery of valuable metals from complex mixtures, reducing the need for virgin material extraction, and mitigating the environmental impacts of conventional mining [13]. Traditionally employed in mining and refining, SX has found increasing relevance in the recovery of metals from secondary sources, such as industrial waste, e‐waste, and end‐of‐life products [14]. The transition toward more sustainable industrial processes is not only a matter of economic necessity but also an urgent environmental imperative [15]. The extraction of metals, particularly through conventional mining methods [16], is a resource‐intensive and environmentally detrimental process that contributes significantly to greenhouse gas (GHG) emissions, habitat destruction, and resource depletion [17]. In this context, SX offers an environmentally friendly alternative, particularly when applied to metal recovery from waste streams, thereby reducing the need for primary resource extraction and mitigating the negative environmental impacts associated with traditional practices [18]. Moreover, the role of SX in driving the circular economy aligns closely with several of the United Nations′ Sustainable Development Goals (SDGs) [19]. Specifically, this review paper is influenced by and seeks to address the following SDGs:

### 1.1. Alignment with SDG 9: Industry, Innovation, and Infrastructure

SDG 9 focuses on building resilient infrastructure, promoting sustainable industrialization, and fostering innovation [20]. SX technology exemplifies these principles by enabling industries to adopt more sustainable practices through the efficient recovery of critical and valuable metals [21]. Innovations in SX have led to the development of more selective and environmentally benign solvents, such as ionic liquids (ILs) and biobased extractant, which reduce the ecological footprint of industrial processes [22]. The implementation of SX within the circular economy supports SDG 9 by facilitating the recovery and reuse of metals, thereby enhancing the sustainability of industrial supply chains [23]. For instance, industries that rely on metals such as copper, nickel, and cobalt can reduce their dependence on primary mining operations by recovering these materials from secondary sources, including e‐waste and industrial residues [24]. This shift not only conserves natural resources but also stimulates innovation in SX techniques, encouraging the development of greener, more efficient methods that align with the goal of sustainable industrialization.

### 1.2. Alignment With SDG 12: Responsible Consumption and Production

SDG 12 emphasizes the need to ensure sustainable consumption and production patterns, particularly through the efficient use of natural resources and the reduction of waste generation [25]. SX plays a critical role in achieving these objectives by promoting the recovery of metals from waste streams, reducing the amount of waste sent to landfills, and contributing to the overall reduction of environmental pollution [26]. One of the key challenges in achieving SDG 12 is managing the growing volume of industrial and e‐waste, which contains valuable metals that are often lost through improper disposal. SX offers a solution by enabling the selective separation and recovery of metals from these complex waste streams, allowing industries to reintroduce these materials into production cycles [27]. For example, the recovery of precious metals such as gold, palladium, and silver from e‐waste not only supports responsible consumption by extending the life cycle of these materials but also reduces the environmental impact of mining and refining operations. The circular economy model, supported by SX, is central to realizing SDG 12′s vision of decoupling economic growth from resource depletion [28]. By improving resource efficiency and minimizing waste, SX helps industries achieve more sustainable production patterns, contributing to a closed‐loop system where materials are continually reused, reducing the need for virgin resources and minimizing environmental degradation [29]. SX systems have demonstrated recovery rates of 85%–95% for a range of metals, such as copper, cobalt, and gold. This high recovery rate contributes directly to the recycling of critical materials, reducing the demand for virgin mining and minimizing resource depletion. SX plays a key role in advancing a circular economy by efficiently recovering metals from waste and secondary resources [30]. For example, in the recovery of rare earth elements (REEs) from e‐waste, SX can contribute to closing the loop on materials that would otherwise be discarded, thus reducing waste generation (SDG 12.5). By improving solvent selectivity and reducing the use of hazardous chemicals, the SX process can minimize the generation of chemical waste, promoting sustainable production practices (SDG 12.4) [31].

### 1.3. Alignment with SDG 13: Climate Action

The extraction and processing of metals are major contributors to global GHG emissions. Conventional mining and refining processes, particularly for metals such as aluminum, copper, and REEs, require significant energy inputs, leading to high levels of carbon emissions. SDG 13, which calls for urgent action to combat climate change and its impacts, highlights the need for industries to adopt more sustainable practices that reduce their carbon footprints [32]. SX, when applied to the recovery of metals from secondary sources, offers a lower energy alternative to traditional extraction methods. By recovering metals from waste materials, SX reduces the need for energy‐intensive mining operations, thereby contributing to a reduction in global carbon emissions [33]. Moreover, recent advancements in the development of greener solvent systems have further minimized the environmental impact of SX processes. For example, the use of ILs as extraction solvents has been shown to significantly reduce the energy requirements of metal recovery, making the process more energy‐efficient and environmentally friendly [34]. In the context of SDG 13, SX supports climate action by enabling industries to reduce their overall carbon emissions while maintaining access to critical materials. The ability to recover metals from waste not only contributes to climate mitigation efforts but also enhances the resilience of supply chains by reducing reliance on geopolitically sensitive primary resources [35]. Moreover, SX not only serves as a tool for improving resource efficiency but also plays a pivotal role in fostering long‐term sustainability across various industrial sectors. By recovering valuable metals from waste streams and reducing the environmental footprint of traditional mining operations, SX contributes significantly to lowering the energy intensity of material processing [36]. This, in turn, directly addresses global concerns surrounding resource scarcity and environmental degradation. Furthermore, as industries increasingly adopt circular economy models, SX is becoming indispensable in critical areas such as urban mining, where e‐waste and other secondary sources are processed to reclaim rare and precious metals. This practice not only conserves natural resources but also aligns with the growing demand for ethical sourcing and sustainable supply chains, particularly in industries such as electronics, renewable energy, and electric vehicles, where the need for critical materials like cobalt, lithium, and REEs is rapidly increasing [37] By advancing the goals of SDG 9 (Industry, Innovation, and Infrastructure), SDG 12 (Responsible Consumption and Production), and SDG 13 (Climate Action), SX holds the potential to drive systemic change in how industries operate within the constraints of environmental and resource sustainability. As the circular economy continues to gain traction, the role of SX will only grow in importance, providing a technological foundation for sustainable industrial practices that balance economic growth with environmental stewardship. Certain advanced SX techniques, such as those using ILs or deep eutectic solvents (DES), can offer reduced energy consumption compared with traditional methods like pyrometallurgical extraction. For example, DES systems have been shown to reduce energy consumption by up to 30%, which directly contributes to reducing carbon emissions (SDG 13.2). [38]. The use of low‐energy extraction processes such as SX can result in significant reductions in GHG emissions, particularly in comparison to more energy‐intensive methods like smelting. We estimate that the potential carbon savings in industrial‐scale applications could reach up to 15% compared with conventional extraction technologies. For example, a study on the SX of copper from e‐waste demonstrated a 70% reduction in energy consumption and a 40% increase in material recovery efficiency when using advanced solvent systems compared with traditional hydrometallurgical methods. These improvements are directly aligned with SDG 12 and SDG 13 targets related to sustainable resource management and climate change mitigation. In this review, we are aimed at comprehensively exploring the advancements, applications, and future directions of SX in the context of the circular economy. Through this lens, we will examine how this technology can enhance resource efficiency and contribute to a more sustainable future, driving innovation across multiple industries. Unlike previous reviews that primarily focus on extraction chemistry or recovery efficiency, this review critically integrates SX technologies with circular economy principles, sustainability assessment, economic feasibility, and emerging digital optimization strategies.

## 2. Methodology of Literature Review

This review was conducted using a structured literature collection and evaluation approach to ensure comprehensive coverage of recent developments in SX technologies and their role in circular economy applications. Relevant scientific literature was systematically collected from internationally recognized databases including Scopus, Web of Science, Google Scholar, and Science Direct. Additional information was obtained from selected books, technical reports, conference proceedings, and policy documents relevant to sustainable metal recovery and circular economy strategies. The literature search primarily focused on publications from 2010 to 2025 in order to capture recent technological advancements, sustainability considerations, and emerging digital approaches associated with SX systems. However, several earlier foundational studies were also included where necessary to provide historical background and fundamental theoretical understanding.

A combination of keywords and Boolean search operators was employed during the literature retrieval process. The main search terms included “solvent extraction,” “metal recovery,” “hydrometallurgy,” “circular economy,” “green solvents,” “ionic liquids,” “metal recycling,” “resource recovery,” “sustainable metallurgy,” “rare earth extraction,” “bio‐based solvents,” “waste valorization,” “artificial intelligence in solvent extraction,” and “digitalization in metallurgical processes.” Different combinations of these keywords were used to maximize coverage and identify relevant interdisciplinary studies.

The inclusion criteria were established to ensure the scientific relevance and quality of the selected literature. Studies were included if they

− focused on SX systems for metal recovery or separation,
− addressed sustainability, environmental impact, or circular economy applications,
− reported extraction efficiency, selectivity, operational performance, or economic aspects,
− discussed industrial applicability or scale‐up potential,
− and were peer‐reviewed and published in reputable scientific journals.

Studies were excluded if they

− were unrelated to metal recovery or SX technologies,
− lacked sufficient technical or methodological detail,
− contained duplicated findings,
− focused solely on unrelated chemical separation systems,
− or were inaccessible in full‐text format.

After the initial literature screening, the selected studies were critically evaluated and categorized according to solvent systems, target metals, extraction mechanisms, environmental implications, operational limitations, economic feasibility, and emerging technological trends. Particular attention was given to comparative analysis among conventional solvent systems, green solvents, ILs, and emerging recovery technologies within the framework of circular economy and sustainable resource management. To improve analytical rigor, the review also incorporated comparative evaluation of recovery efficiencies, environmental performance, scalability, and sustainability trade‐offs reported across different studies. Variability in experimental conditions, operational scales (laboratory, pilot, and industrial), and methodological approaches was considered during data interpretation to minimize overgeneralization and improve contextual understanding of reported findings. Furthermore, the review emphasizes research gaps, technological limitations, and future directions associated with SX systems, particularly regarding solvent recyclability, environmental safety, industrial scalability, economic viability, and integration of artificial intelligence and digital optimization tools for sustainable metal recovery processes.

## 3. Current Advancements in SX

Recent advancements in SX technology have significantly enhanced the efficiency, selectivity, and sustainability of metal recovery processes. Traditionally, SX has been a cornerstone of hydrometallurgical operations, particularly in the separation and purification of base and precious metals. However, as environmental concerns and the need for sustainable practices grow, researchers and industries are shifting toward more eco‐friendly solvents and innovative methodologies that align with circular economy principles.

### 3.1. Key Solvent Systems

SX relies on a variety of organic solvent systems that are specifically designed to selectively extract target metals from aqueous solutions. Among the most widely used are quaternary ammonium salts, organophosphorus compounds, and more recently, DES as greener alternatives. Each of these systems has been optimized for particular metal groups and operates based on differing physicochemical interactions [39]. Aliquat 336, a quaternary ammonium salt, is well‐known for its ability to extract precious metals such as gold and platinum group elements due to its high selectivity and stability under acidic conditions. However, its high cost and issues with viscosity in large‐scale operations present challenges [40]. Cyanex 272, an organophosphinic acid derivative, is another industrially important extractant particularly effective in separating cobalt from nickel. Its strong selectivity has made it a cornerstone in hydrometallurgical applications, but concerns remain regarding its toxicity and limited biodegradability [41]. Another extensively used extractant is D2EHPA (di‐(2‐ethylhexyl) phosphoric acid), which demonstrates high efficiency in extracting uranium, zinc, and REEs. Despite its performance, it can form stable emulsions, complicating phase separation and reducing operational efficiency [42]. Tributyl phosphate (TBP) is predominantly used in nuclear fuel reprocessing, especially for the separation of uranium and plutonium. It offers excellent extraction efficiency but suffers from flammability and long‐term degradation issues. Recent developments have focused on more sustainable options, including choline chloride–based DES. These solvents are considered green due to their low toxicity, biodegradability, and tenability [43]. DES has shown promise in extracting metals like copper, nickel, and REEs from both primary and secondary sources. However, their extraction efficiency can be lower compared with conventional solvents, and their long‐term stability under industrial conditions is still under investigation. Overall, although traditional solvent systems have played a crucial role in advancing SX technologies, they are increasingly being scrutinized for their environmental and health impacts. Table 1 summarizes the most commonly used solvent systems in SX [10].

**Table 1:** Most commonly used solvent systems in solvent extraction (SX) with an overview of target metal, advantages and drawbacks.

Solvent system	Target metals	Advantages	Drawbacks	References
Aliquat 336 (quaternary ammonium salt)	Gold, platinum group metals, rare earth elements	High selectivity; stable under acidic conditions	High cost; viscosity issues in scale‐up	[1, 2]
Cyanex 272 (phosphinic acid derivative)	Cobalt, nickel, zinc	Excellent Co/Ni separation; widely used	Poor biodegradability; acidic waste generation	[3, 4]
Choline chloride–based DES	Copper, nickel, rare earth elements	Low toxicity; biodegradable; customizable properties	Lower efficiency in some cases; stability issues	[5–7]
D2EHPA (Di‐(2‐ethylhexyl) phosphoric acid)	Uranium, zinc, rare earth elements	High loading capacity; scalable	Tendency for emulsion formation; acidity requirement	[8, 9]
TBP (tributyl phosphate)	Uranium, plutonium	Excellent for actinide separation in nuclear industry	Flammability; degradation over time	[10]

Table 1 shows that conventional solvent systems offer high extraction efficiency and selectivity, but often suffer from environmental and regeneration issues. In contrast, greener alternatives improve sustainability but may have lower efficiency or higher cost. Overall, the table highlights a clear trade‐off between performance and environmental impact, indicating that no single solvent system is universally optimal.

### 3.2. Comparative Evaluation of SX With Alternative Metal Recovery Technologies

Although SX has become a widely adopted technique for selective metal recovery and purification, its effectiveness should be critically evaluated alongside other metal recovery technologies used in circular economy systems. Alternative approaches such as pyrometallurgical processing, bioleaching, adsorption‐based separation, and membrane technologies have also been extensively explored for recovering valuable metals from primary and secondary resources. Pyrometallurgical techniques offer high processing efficiency and rapid metal recovery; however, they are frequently associated with high energy consumption, significant GHG emissions, and operational challenges when processing low‐grade ores or complex waste streams. In contrast, SX operates under relatively mild conditions and provides higher selectivity for targeted metal separation, making it more suitable for hydrometallurgical recycling systems. Bioleaching has emerged as an environmentally favorable approach due to its lower chemical and energy requirements. Nevertheless, slow reaction kinetics, microbial sensitivity, and scalability limitations continue to restrict its broader industrial implementation. Compared with bioleaching, SX demonstrates greater process efficiency and industrial adaptability. Adsorption and membrane‐based separation technologies also show potential for selective metal recovery. However, limitations such as adsorbent regeneration difficulties, membrane fouling, and reduced selectivity in multimetal systems remain significant challenges. SX, despite concerns related to solvent loss and toxicity, continues to offer strong advantages in terms of selectivity, scalability, and integration with established hydrometallurgical operations. These comparisons indicate that SX remains a highly competitive technology for circular economy‐driven metal recovery; however, future advancements should focus on improving solvent sustainability, reducing environmental impacts, and integrating hybrid recovery approaches for enhanced resource efficiency.

Table 2 demonstrates that SX offers high selectivity and operational flexibility compared with alternative metal recovery technologies. However, pyrometallurgical processes generally achieve higher throughput, whereas bioleaching and emerging electrochemical methods provide better environmental performance but suffer from slower kinetics and scalability limitations. Overall, the comparison highlights that no single technology is universally superior, and the choice depends on a balance between efficiency, environmental impact, and industrial feasibility within circular economy systems.

**Table 2:** Comparative evaluation of solvent extraction and alternative metal recovery technologies in circular economy applications.

Technology	Advantages	Limitations	Suitability for circular economy
Solvent extraction	High selectivity, scalable	Solvent toxicity, solvent loss	High
Pyrometallurgy	Fast processing	High energy demand	Moderate
Bioleaching	Environmentally friendly	Slow kinetics	Moderate
Adsorption	Simple operation	Regeneration challenges	Moderate
Membrane separation	Low chemical use	Membrane fouling	Moderate

### 3.3. Development of Green Solvents

One of the most significant trends in SX is the development of green solvents, which are aimed at replacing traditional organic solvents that often pose environmental and health risks [44]. Green solvents include ILs, DES, and biobased solvents, which offer a range of benefits including lower toxicity, higher biodegradability, and improved recyclability [45]. ILs have garnered considerable attention due to their tunable properties, nonvolatility, and ability to dissolve a wide range of metals [46]. Their potential to enhance the selectivity and efficiency of metal extraction while reducing solvent losses makes them highly attractive in sustainable SX processes [47]. DES are emerging as an alternative to ILs due to their cost‐effectiveness and low environmental impact [48]. Composed of natural or biodegradable components, DES can offer competitive extraction efficiency for metals like copper, zinc, and REEs, without the environmental drawbacks of conventional organic solvents [49]. Biobased solvents, derived from renewable resources such as plant‐based materials, provide another promising avenue [50]. Their low toxicity and renewable nature make them suitable for sustainable SX processes, particularly in industries that require environmentally responsible solutions [51]. Table 2 provides a quick reference for comparing the performance and economic aspects of various SX methods, drawn from case studies and published data. The solvents listed include ILs, DES, organic solvents, and aqueous solutions, and the table reflects their extraction efficiencies and the cost per kilogram of recovered metal. This allows for a more quantitative and systematic comparison of the different solvent systems utilized in both primary mining and recycling applications.

Table 3 highlights a clear relationship between extraction efficiency and economic cost across different solvent systems used in metal recovery. High‐performance solvents generally achieve greater extraction efficiencies but are associated with increased cost per kilogram of recovered metal, limiting their economic feasibility for large‐scale applications. In contrast, lower cost solvents tend to exhibit reduced efficiency, indicating a trade‐off between performance and affordability. This demonstrates that solvent selection in circular economy applications cannot be based solely on efficiency, but must also consider process economics and scalability.

**Table 3:** Comparisons of different solvents with their extraction efficiencies and cost per kilogram of recovered metal.

Solvent type	Target metal(s)	Extraction efficiency (%)	Cost per Kg recovered (USD)	Source/reference
Ionic liquids	Copper, nickel, cobalt	85%–95%	5.60	46
Deep eutectic solvents (DES)	Rare earth elements (REEs), gold	90%	12.50	48
Organic solvents	Gold, silver, palladium	75%–80%	15.00	49
Aqueous solutions	Zinc, copper, uranium	95%	3.40	50

### 3.4. Advancements in Process Optimization

In addition to the development of greener solvents, advancements in process optimization have played a key role in improving the efficiency and scalability of SX techniques [52]. Innovations such as microfluidic extraction systems, continuous extraction processes, and membrane‐based SX have enhanced metal recovery rates, reduced solvent usage, and minimized waste generation. Microfluidic extraction systems offer enhanced control over reaction conditions, allowing for more precise metal separation in small volumes, which is particularly advantageous in high‐value applications like pharmaceutical and electronics industries. These systems not only improve the selectivity of metal extraction but also reduce solvent and energy consumption [53]. Continuous SX processes have revolutionized the scalability of SX, especially in industrial applications. Unlike batch extraction, continuous processes enable the uninterrupted flow of feedstock and solvents, leading to higher throughput and more efficient use of resources. This is especially important in large‐scale operations where SX is integrated with other refining or recycling processes [54]. Membrane‐based SX combines the selectivity of SX with the separation efficiency of membrane technologies. This hybrid approach allows for the selective removal of specific metals from complex solutions while minimizing solvent losses and waste generation. The use of membranes can also improve the energy efficiency of the extraction process, further aligning it with sustainability goals [55].

### 3.5. Industrial Applications

SX remains widely used in a range of industries, from mining to recycling. However, recent advancements have expanded its applications to more specialized and critical areas, including the recovery of REEs, critical materials and noble metals. REE recovery has become a focal point of SX research due to the increasing demand for these elements in the production of high‐tech devices, electric vehicles, and renewable energy technologies [56]. Recent advancements have focused on improving the selectivity of solvent systems for the separation of REEs from complex matrices, particularly from e‐waste and other secondary sources [57]. Critical materials, such as lithium, cobalt, and nickel, are essential for modern industries, including battery manufacturing and electronics. The growing push toward electrification and renewable energy has led to heightened demand for these materials, making SX a vital tool in their recovery from both primary ores and recycled materials [58]. Noble metals, including gold, platinum, and palladium, are increasingly recovered through SX, particularly from secondary sources like e‐waste. New solvent systems are being developed to improve the selectivity and yield of these valuable metals while minimizing the use of hazardous chemicals traditionally employed in their extraction [59]. These advancements collectively position SX as a critical technology in achieving the objectives of the circular economy. By improving the sustainability and efficiency of metal recovery processes, SX not only conserves resources but also reduces the environmental impacts associated with mining and refining, making it an indispensable tool in modern industrial practices.

### 3.6. Integration of Low‐Energy Catalytic Processes for Enhanced Sustainability

One of the key challenges in advancing SX technologies within the framework of the circular economy is the high energy demand often associated with conventional separation and reaction processes. Recent developments in coordination chemistry have opened new avenues for designing low‐energy catalytic systems that align with sustainability goals. As coordination‐driven innovations enable catalytic transformations under milder conditions, significantly reducing the energy footprint of chemical production. [60] These low‐energy approaches rely on the strategic design of ligand environments and metal centers to facilitate efficient reaction pathways with minimal thermal input. Incorporating such coordination‐based strategies into SX processes—either by modifying extractants or integrating catalytic cycles—holds promise for enhancing the sustainability and scalability of metal recovery and recycling systems. This direction not only supports energy efficiency but also complements the broader goals of green chemistry and industrial decarbonization [61].

## 4. Role of SX in Enhancing Circular Economy Practices

In the context of the circular economy, SX has emerged as a key process for closing material loops, reducing waste, and improving the sustainability of resource‐intensive industries. As the global economy transitions from a linear “take–make–dispose” model to a more circular one, SX is becoming crucial in various stages of resource recovery, particularly in metal recycling and urban mining. This section explores the role of SX in enhancing circular economy practices by enabling the efficient recovery, reuse, and recycling of metals, thereby contributing to resource conservation and environmental sustainability.

### 4.1. Facilitating Metal Recovery From Waste Streams

One of the most impactful contributions of SX to the circular economy is its ability to recover valuable metals from waste streams. These waste streams can come from industrial processes, e‐waste, or even municipal solid waste, where significant amounts of recoverable metals are often discarded. SX offers a highly efficient method for isolating and purifying metals from complex waste matrices, allowing them to be reintroduced into the supply chain. E‐waste, for instance, is a rapidly growing concern, with millions of tons generated globally every year. E‐waste contains precious and rare metals such as gold, silver, platinum, and REEs, all of which can be recovered through SX. By processing e‐waste and recovering these metals, SX not only reduces the need for primary mining operations but also mitigates the environmental and human health risks associated with improper e‐waste disposal. Industrial waste streams also present a valuable source of recoverable metals, particularly in sectors like manufacturing, mining, and refining. Spent catalysts, slag, and other industrial by‐products often contain residual metals that can be extracted through SX processes. This reduces the environmental burden of waste disposal and provides a more sustainable alternative to traditional landfilling or incineration [62].

### 4.2. Supporting Sustainable Supply Chains

SX plays a pivotal role in creating more sustainable and resilient supply chains by recovering critical materials and metals that are essential for industries like electronics, renewable energy, and automotive manufacturing. As industries increasingly shift toward sustainable sourcing and closed‐loop systems, SX becomes an integral part of ensuring the availability of critical raw materials through recycling rather than relying solely on primary mining. REEs are a prime example of critical materials whose supply chains are vulnerable to geopolitical risks and environmental concerns. SX allows for the recovery of REEs from secondary sources, such as e‐waste, magnets, and industrial residues. By integrating SX into the recycling of these materials, industries can reduce their dependence on primary mining operations, which are often associated with significant environmental degradation [63]. Similarly, metals like cobalt, nickel, and lithium, which are critical for battery production in electric vehicles and renewable energy storage systems, can be recovered through SX from spent batteries and electronic devices. This contributes to the circular economy by ensuring that these finite resources are reused rather than discarded, supporting the transition to a greener and more sustainable energy future [64].

### 4.3. Reducing Environmental Impact and Resource Depletion

In addition to improving material recovery, SX helps to significantly reduce the environmental impact of metal recovery processes. By enabling the recycling and reuse of metals, SX reduces the demand for primary extraction, which is often associated with severe environmental consequences such as habitat destruction, water pollution, and high energy consumption [65]. Reducing the need for primary mining operations, particularly for metals like copper, nickel, and REEs, are resource‐intensive and environmentally damaging. By recovering these metals from waste streams through SX, the need for new mining projects is reduced, leading to lower energy consumption, fewer GHG emissions, and less disturbance to ecosystems [66]. Minimizing waste generation in SX processes can be designed to recover metals with high efficiency, minimizing the amount of residual waste that is generated during the recycling process. In many cases, the by‐products of SX can be treated or repurposed, further contributing to waste reduction efforts within the circular economy.

### 4.4. Comparative Assessment of SX Systems

SX technologies exhibit varying degrees of scalability, economic performance, and environmental sustainability depending on the solvent system and application context [67]. Traditional organic solvents such as kerosene‐based diluents and organophosphorus compounds (e.g., Cyanex 272) are well‐established in industrial processes due to their high selectivity and efficiency. However, their environmental and health hazards, coupled with regulatory pressure, have shifted attention toward greener alternatives. ILs and DESs offer tunable properties, high thermal stability, and reduced volatility, making them promising candidates for sustainable metal recovery [68]. Nonetheless, their economic viability remains constrained by high synthesis costs and uncertainties in long‐term toxicity and degradation behavior. Moreover, life cycle assessments (LCAs) of IL‐based systems reveal mixed results depending on the precursors used and solvent recycling efficiency. From a techno‐economic perspective, organic solvents remain cost‐effective at scale, particularly in systems where solvent loss is minimized through closed‐loop recycling. In contrast, DESs are emerging as more sustainable options due to their low cost, bioderived components, and ease of synthesis. However, further industrial validation is required to confirm their scalability and metal selectivity across various feedstocks [69]. Several comparative LCA and techno‐economic studies suggest that DES‐based systems can reduce CO_2_ emissions by up to 30%–40% compared with traditional systems, provided solvent recovery rates exceed 80%. Table 3 summarizes key attributes of different SX systems across critical performance indicators.

Table 4 provides a comparative overview of selected SX systems, highlighting differences in extraction performance, selectivity, environmental compatibility, and operational feasibility. The comparison indicates that conventional solvent systems generally offer strong extraction efficiency and well‐established industrial applicability, whereas emerging green and alternative solvents demonstrate improved environmental performance but may face limitations in stability, cost, or scale‐up readiness. These results emphasize that SX systems cannot be evaluated on a single performance metric; instead, a multicriteria balance involving efficiency, sustainability, and process economics is essential for circular economy applications.

**Table 4:** Comparative overview of selected solvent extraction systems.

Solvent system	Target metals	Recovery efficiency (%)	Estimated cost (USD/kg metal)	Recyclability (%)	CO_2_ savings versus. traditional (%)	Industrial readiness
Kerosene + Cyanex 272	Co, Ni, REEs	90–95	2.0–3.5	~75	Baseline	High
Ionic liquids (e.g., [C4mim]PF6)	Li, rare earth elements	85–92	5.0–10.0	~60	15–25	Medium
DES (choline chloride + urea)	Zn, Cu, Cr	80–88	1.5–2.5	~85	30–40	Low–medium
Supercritical CO_2_ + ligands	Au, Pd	75–85	8.0–12.0	~50	35–45	Experimental

### 4.5. Life Cycle and Toxicological Considerations of Green Solvents

Although ILs and DES are often presented as environmentally friendly alternatives to traditional organic solvents, their green credentials must be critically evaluated. The synthesis of some ILs can involve toxic precursors, complex purification steps, and high energy input, potentially offsetting their benefits [70]. Moreover, concerns have been raised regarding their environmental persistence, biodegradability, and long‐term ecotoxicological effects. Recent studies have highlighted that certain ILs may exhibit toxicity toward aquatic organisms and may not degrade easily in natural environments, raising questions about their suitability for large‐scale applications without proper end‐of‐life management strategies [71] Similarly, although DES are often derived from natural or biodegradable components, their environmental behavior depends on specific compositions and concentrations. LCAs of ILs and DES are increasingly being used to evaluate cradle‐to‐grave impacts, accounting for synthesis, use, degradation, and disposal. These insights underscore the importance of a balanced approach, where the selection and application of green solvents are guided by comprehensive sustainability criteria, including toxicity, biodegradability, and regulatory frameworks [72]. A more nuanced understanding of these factors is critical to ensuring that the deployment of green solvents genuinely supports the principles of green chemistry and circular economy. The full life cycle of SX in assessing its environmental impact, which evaluate the end of life impacts, such as solvent degradation, waste generation, and recycling rates emphasizes the need for regulatory measures to ensure that SX processes are not only energy‐efficient but also sustainable throughout their life cycle, from synthesis of solvents to their disposal or reuse. [73].

### 4.6. Case Studies in Circular Economy Applications

Several industries have successfully integrated SX into their circular economy strategies, demonstrating the feasibility and benefits of this approach. For example, the urban mining of e‐waste, particularly in countries with high technological turnover, has emerged as a critical area where SX plays a central role in recovering valuable metals. In Japan, innovative urban mining initiatives are using SX to recover gold and REEs from discarded electronic devices. These recovered materials are then reintroduced into manufacturing processes, reducing the country′s reliance on imported raw materials [74]. In the case of Japan′s REE recovery from e‐waste, SX helped reduce CO_2_ emissions by 15%–20% compared with conventional methods. This reduction was largely due to energy‐efficient extraction techniques and higher solvent recovery rates, which contributed to both energy savings and reduced material waste [75]. For example, the Hitachi Group in Japan has implemented SX processes in REE recovery from end‐of‐life electronics. Reported yields in pilot‐scale trials exceeded 90% for selected REEs, with solvent recovery efficiencies around 85%, contributing to a significant reduction in raw material import dependence [76]. The European Union has also implemented policies aimed at promoting the circular economy, particularly in the recycling of critical raw materials. SX is being used in pilot projects to recover valuable metals from waste products, such as batteries and industrial residues, thus aligning with the EU′s broader sustainability and resource conservation goals [77]. In Europe, hydrometallurgical systems utilizing SX for copper and zinc recycling demonstrated a 20%–25% reduction in carbon emissions compared with conventional smelting operations. These reductions were a result of the lower temperatures required for SX, as well as improvements in solvent reuse and energy optimization (European Commission, 2023). In the European context, the ProSUM (Prospecting Secondary raw materials in the Urban mine and Mining wastes) project funded under Horizon 2020 provided comprehensive data on secondary metal flows and supported the deployment of SX methods in pilot e‐waste processing plants [78]. Cost‐benefit analyses from ProSUM indicated a potential 20%–30% reduction in processing costs when using optimized solvent systems, alongside improvements in selectivity and environmental performance (ProSUM Final Report, 2020). In the United States, industries are utilizing SX to reclaim metals from industrial by‐products and waste streams, particularly in the energy sector. This approach helps reduce the environmental footprint of energy production and supports the recycling of metals like uranium and thorium from nuclear waste, which are then reused in energy generation [79]. These examples illustrate how SX can be applied at various stages of the supply chain to promote the circular economy, reduce environmental impact, and enhance resource efficiency [80]. These examples illustrate the practical feasibility and growing interest in SX technologies as part of circular economy strategies, but they also underscore the need for ongoing evaluation of scale‐up performance, solvent recyclability, and market integration.

### 4.7. Carbon Footprint Considerations in SX

SX is typically evaluated based on recovery efficiency and selectivity, but its potential to significantly reduce GHG emissions within circular economy frameworks is increasingly recognized. Conventional primary metal extraction, including mining and pyrometallurgical processing, is highly energy‐intensive and contributes substantially to global carbon emissions. By contrast, SX applied to secondary raw materials—such as e‐waste, spent batteries, and industrial residues—can offer notable carbon savings due to reduced energy consumption, shorter processing chains, and lower raw material requirements [81]. For example, a LCA study on REE recovery from end‐of‐life magnets found that SX‐based processes could reduce CO_2_ emissions by approximately 60% compared with primary mining routes. These reductions largely result from eliminating energy‐intensive steps such as ore crushing, roasting, and leaching. Additionally, solvent recycling within these processes further decreases emissions associated with solvent production, underscoring the importance of solvent reuse in minimizing overall carbon footprints. Similarly, hydrometallurgical recovery of lithium and cobalt from spent lithium‐ion batteries often incorporates SX for metal separation and purification. It was reported that such processes can achieve carbon emission reductions of 30%–50% compared with virgin ore processing [82]. The greatest savings were observed when SX was coupled with renewable energy inputs and solvent regeneration methods, highlighting the significance of integrated system design for enhanced sustainability. In an industrial context, the EU‐funded EcoMetals project (2023) evaluated SX of copper from metallurgical slags and industrial effluents, revealing potential carbon savings of up to 40% relative to conventional smelting and refining. The project also identified that further optimization of solvent selectivity and recycling could improve environmental outcomes even more [83]. Despite these promising examples, SX technologies currently lack standardized carbon benchmarking metrics. Variability in solvent chemistry, feedstock composition, and process configurations complicate direct comparisons across different systems. To address this challenge, emerging methodologies that integrate cradle‐to‐grave LCA with process simulation tools are being developed [84], enabling more accurate quantification of emissions savings and supporting better informed decision‐making for stakeholders and policymakers. Overall, integrating SX into circular metal recovery systems offers a promising pathway to reduce the GHG emissions associated with resource extraction. Continued advancements in solvent design, process integration, and the adoption of renewable energy sources will be critical to maximizing carbon savings. Establishing robust carbon footprint benchmarks for SX technologies will be essential to validate environmental benefits and promote their broader adoption within sustainable resource management frameworks [85].

## 5. Environmental and Economic Benefits

SX offers numerous environmental and economic benefits that align with the principles of the circular economy. By enhancing the recovery of metals and minimizing waste, this technology not only contributes to resource conservation but also supports sustainable industrial practices. This section will explore the key quantified environmental advantages and economic implications of implementing SX processes in various industries.

### 5.1. Environmental Benefits

#### 5.1.1. Reduction of Waste Generation

One of the most significant environmental benefits of SX is its ability to reduce waste generation. Traditional metal extraction processes often result in large amounts of waste, including tailings and slag, which can pose significant environmental hazards. In contrast, SX‐based systems can recover up to 90%–95% of valuable metals from secondary sources, such as e‐waste and industrial effluents, thereby minimizing residual waste [86]. For instance, SX applied to printed circuit board leachates has shown a waste volume reduction of over 70% compared with landfilling or incineration processes [87]. By minimizing waste, SX helps mitigate the environmental impacts associated with waste disposal, such as soil contamination, water pollution, and habitat destruction. Additionally, the ability to recover metals from secondary sources reduces the pressure on landfills and waste management systems, supporting more sustainable waste management practices.

#### 5.1.2. Lowering GHG Emissions

The extraction and processing of metals typically involve significant energy consumption, resulting in substantial GHG emissions. SX, on the other hand, often requires less energy compared with traditional mining and refining methods. [88]. By improving the efficiency of metal recovery, SX contributes to lower carbon footprints across various industrial sectors. For example, SX‐based recovery of copper from waste streams emits 30%–50% less CO_2_ equivalent per kg of metal than traditional mining and smelting processes. In e‐waste recycling scenarios, the carbon footprint is further reduced due to the elimination of energy‐intensive ore beneficiation and smelting stages. This makes SX a critical contributor to decarbonizing the metal production value chain. This reduction in energy consumption directly correlates with lower GHG emissions, making SX a more environmentally friendly option for metal recovery [89].

#### 5.1.3. Reduction in Water Usage

Water usage is another crucial metric in assessing environmental performance. SX systems, particularly those integrated into closed‐loop hydrometallurgical circuits, have demonstrated water savings of 35%–45% compared with primary extraction processes [90]. This efficiency is achieved through the reuse of aqueous and organic phases in the SX cycle, along with reduced reliance on high‐volume leaching operations. Water‐efficient SX designs are especially beneficial in regions facing freshwater scarcity. In addition to energy savings and waste reduction, SX also demonstrates favorable water efficiency. Traditional leaching and flotation processes used in mining operations can consume 2–4 m^3^ of water per ton of ore processed, often resulting in heavily contaminated effluents. In contrast, SX circuits—especially when operated in closed or semiclosed loops—can reduce freshwater consumption by up to 60%, as solvents are often regenerated and reused within the system. For instance, a comparative study of SX‐based zinc recovery from industrial effluents used less than half the process water required by equivalent hydrometallurgical methods [91]. This makes SX especially valuable in water‐scarce regions and for industries aiming to reduce their freshwater footprint.

#### 5.1.4. Conservation of Natural Resources

SX plays a critical role in conserving natural resources by promoting the recycling and reuse of metals. By recovering valuable metals from waste streams, industries can reduce their dependence on primary raw materials, thereby alleviating the pressure on finite natural resources [92]. This conservation is particularly important for metals that are becoming increasingly scarce due to rising demand and environmental regulations. SX provides a sustainable solution for extending the lifespan of these resources and ensuring their availability for future generations. For instance, the use of SX in recovering REEs and lithium from end‐of‐life products has reduced the demand for new mining operations by up to 25% in some pilot projects. Such practices support resource circularity and long‐term sustainability goals. Furthermore, the European UrbanMine initiative (2022) estimated that the implementation of SX in urban mining systems could reduce the demand for virgin metal ores by up to 40%. [93]. This contributes to sustainable resource management and ensures the long‐term availability of strategically important elements.

### 5.2. Economic Benefits

#### 5.2.1. Cost Savings and Resource Efficiency

The economic benefits of SX are closely tied to its ability to enhance resource efficiency. By recovering valuable metals from waste materials, industries can realize significant cost savings compared with sourcing raw materials from traditional mining operations. Implementing SX processes can significantly reduce operational costs by minimizing the reliance on extensive mining, transportation, and raw material processing, making it particularly advantageous in industries affected by volatile metal prices. This approach offers a stable, cost‐effective alternative while enhancing profitability by enabling companies to recover high‐value metals from waste streams [94] For example, the hydrometallurgical approach, including SX, has been found to significantly reduce energy consumption and carbon emissions. According to SX in urban mining, scenarios can cut carbon emissions by up to 30% when compared with primary mining processes. This is primarily due to lower energy requirements in SX compared with energy‐intensive methods like Pyrometallurgy [95]. The added revenue potential from these recovered metals contributes to improved financial performance, positioning SX as a valuable strategy for both economic and operational efficiency.

#### 5.2.2. Job Creation and Economic Growth

The adoption of SX technologies can also drive job creation and economic growth in the regions where these processes are implemented. As industries transition to more sustainable practices, new job opportunities can arise in areas such as research and development, operations, and waste management. Investment in SX technologies can drive economic growth by fostering innovation and supporting the development of sustainable industries, enabling the creation of new businesses and initiatives focused on recycling and resource recovery, which strengthens local economies [96]. Additionally, integrating SX into metal recovery processes enhances supply chain resilience, reducing vulnerability to market fluctuations and geopolitical risks, thereby ensuring a steady supply of critical materials and supporting long‐term economic stability. The environmental and economic benefits of SX underscore its vital role in advancing the circular economy. By enhancing resource efficiency, reducing waste, and lowering GHG emissions, SX not only contributes to environmental sustainability but also supports the economic viability of industries across multiple sectors. As the demand for metals continues to rise, particularly in the context of renewable energy and high‐tech applications, SX will play an increasingly important role in ensuring the sustainable recovery and management of these essential resources.

### 5.3. Benchmarking SX Against Alternative Recovery Methods

SX remains one of the most industrially adopted separation techniques for metal recovery. To better understand the relative strengths and limitations of SX, it is essential to benchmark it against alternative recovery technologies that are gaining attention in the field of e‐waste recycling and urban mining [97]. These include bioleaching, supercritical CO_2_ extraction, and electrochemical recovery. Bioleaching employs microorganisms to selectively solubilize metals. It is environmentally benign and energy‐efficient but generally slower and less predictable than SX. Recovery rates for bioleaching of precious metals such as gold and copper can reach up to 90% under optimized conditions, but process control and microbial sensitivity remain major limitations [98]. Additionally, the low TRL and long residence times hinder industrial scalability. Supercritical CO_2_ extraction, particularly when combined with chelating agents, presents a green and selective alternative. However, its application to metal recovery is still largely experimental, with limited large‐scale data [99]. The high‐pressure equipment and operational complexity also contribute to higher capital costs, although the solvent‐free nature and potential for high selectivity are notable advantages. Electrochemical recovery offers high metal specificity and the potential for direct metal deposition. It is especially effective for recovering metals like copper and gold in high concentrations [100]. However, electrochemical processes often suffer from high energy demands, and their efficiency can be compromised when dealing with complex multimetal waste streams without pretreatment—often necessitating prior SX or leaching steps. Compared with these alternatives, SX offers a more mature technological platform (TRL 7–9), with high recovery efficiencies (> 95% for many metals), established scalability, and adaptability across various feedstocks [101]. However, its environmental footprint depends heavily on solvent choice and process design.

Table 5 presents a benchmarking of major metal recovery technologies, showing clear differences in efficiency, environmental impact, cost, and scalability. Pyrometallurgical methods generally achieve high processing capacity but are associated with high energy consumption and carbon emissions. Hydrometallurgical and SX‐based approaches offer improved selectivity and lower energy requirements, but their performance is often limited by chemical usage and waste management concerns. Emerging technologies such as bioleaching and electrochemical recovery demonstrate strong environmental advantages, yet they remain constrained by slower kinetics and industrial scalability challenges. Overall, the benchmarking reveals that no single technology fully satisfies all circular economy requirements, reinforcing the need for integrated and hybrid recovery strategies.

**Table 5:** Benchmarking of metal recovery technologies.

Technology	Target metals	Recovery efficiency (%)	Cost estimate (USD/kg)	Timescale	Industrial Readiness	Environmental impact
Solvent extraction (SX)	REEs, Co, Ni, Cu	85–95	2–5	Hours	High	Moderate
Bioleaching	Cu, Au, Zn	60–90	1–3	Days–weeks	Medium	Low
Supercritical CO_2_	Au, Pd, Pt	70–85	8–12	Hours–days	Low–medium	Low–moderate
Electrochemical recovery	Cu, Ag, Zn	80–98	3–7	Hours–days	Medium	Depends on electricity source

## 6. Challenges and Opportunities for Innovation

Although SX presents numerous advantages in promoting sustainability and resource efficiency, several challenges need to be addressed to optimize its implementation within the circular economy. This section explores the key challenges associated with SX technologies and highlights opportunities for innovation to enhance their effectiveness and sustainability.

### 6.1. Key Challenges

#### 6.1.1. Solvent Selection and Environmental Impact

The choice of solvents used in extraction processes is critical to their environmental sustainability. Many conventional solvents are toxic, volatile, and pose risks to human health and the environment. This necessitates a careful assessment of the environmental impact of solvents and the need for alternative, greener options. Although advancements in green solvents such as ILs and DES are promising, their long‐term environmental effects and potential toxicity need thorough evaluation. Ensuring that these solvents are not only effective in metal recovery but also environmentally benign is essential for sustainable practice. The use of certain solvents may be subject to strict regulatory frameworks, impacting their adoption in industrial applications. Companies must navigate these regulations while ensuring compliance, which can complicate the implementation of SX technologies. In addition to regulatory restrictions, solvent degradation and waste management further complicate sustainable implementation [102]. During extraction processes—particularly under acidic, oxidative, or high‐temperature conditions—solvents may undergo chemical degradation, forming by‐products that are difficult to detect, potentially hazardous, and environmentally persistent. These degradation products can contribute to soil and water contamination, requiring additional treatment or disposal protocols that may be energy‐intensive or costly. Moreover, although emerging solvent systems such as ILs and DESs offer significant advantages, their real‐world applications are often constrained by challenges in solvent recovery and recyclability. Over multiple extraction cycles, ILs may accumulate impurities, undergo structural breakdown, or exhibit increased viscosity, reducing their effectiveness and necessitating frequent replacement. The regeneration and purification of these solvents can be technically demanding, require specialized equipment, and, in some cases, may negate the environmental gains of their use. These challenges are compounded by the evolving regulatory landscape. Many jurisdictions have implemented or are in the process of drafting legislation to limit or phase out the use of certain solvents, particularly halogenated or petrochemical‐based variants [103]. For example, chlorinated solvents like trichloroethylene and dichloromethane are increasingly restricted due to their toxicity and environmental persistence. This creates uncertainty for industries seeking to adopt new technologies and underscores the need for solvent systems that are both high‐performing and compliant with international chemical safety standards. Therefore, solvent selection should not only prioritize extraction efficiency but must also reflect a holistic understanding of environmental impact, degradation behavior, end‐of‐life management, and regulatory viability. A LCA approach is essential in evaluating the overall sustainability of SX systems, ensuring alignment with green chemistry principles and circular economy strategies [104]. Future research should also focus on the development of robust, recyclable, and less toxic solvent systems that can meet the dual demands of industrial performance and ecological responsibility.

#### 6.1.2. Process Optimization and Scale‐Up

Scaling up SX processes from laboratory settings to industrial applications poses significant challenges. Many innovative SX techniques may work effectively on a small scale but encounter difficulties when applied at larger scales due to factors such as efficiency, cost, and process stability. Optimizing SX processes for large‐scale operations can involve considerable investment in technology and infrastructure. This raises concerns regarding the economic feasibility of implementing these technologies in various industries, especially for small‐ and medium‐sized enterprises (SMEs). Ensuring consistent performance in SX processes can be challenging due to variations in feedstock composition, solvent properties, and operational conditions. Developing robust and reliable processes is essential to maintain efficiency and effectiveness in metal recovery.

#### 6.1.3. Competition from Alternative Technologies

SX competes with various other metal recovery technologies, such as pyrometallurgy and hydrometallurgy, as well as emerging methods like bioleaching and electrochemical extraction. Each method has its own advantages and limitations, creating competition in terms of efficiency, cost, and environmental impact. The rapid evolution of alternative technologies can create uncertainty for SX, particularly if these alternatives prove to be more economically viable or environmentally friendly. The industry must adapt to these dynamics to maintain its relevance in the metal recovery landscape. For instance, biohydrometallurgical techniques—such as bioleaching and biosorption—have gained traction due to their ability to selectively recover metals using microorganisms while minimizing energy consumption and chemical inputs [105]. Integrating these biological methods with SX processes may offer synergistic benefits, enhancing overall sustainability and circular economy outcomes. Additionally, comprehensive LCA frameworks tailored for circular recovery systems provide critical insights into the environmental trade‐offs of competing technologies. A critical evaluation of LCA tools applied to material recovery systems, underlining the need for holistic environmental metrics in evaluating recovery technologies. Their framework supports decision‐making processes aimed at selecting the most sustainable strategies in circular hydrometallurgy. Incorporating these perspectives into SX strategies can greatly enhance the environmental and resource efficiency of circular recovery models [106].

### 6.2. Opportunities for Innovation

Despite these challenges, there are significant opportunities for innovation in SX technologies that can help overcome existing limitations and enhance their integration into circular economy practices.

#### 6.2.1. Advancements in Green Solvent Development

Continued research and development in green solvents can lead to the discovery of new, more effective, and environmentally friendly options for metal extraction. The adoption of biodegradable and renewable biobased solvents in SX processes can significantly reduce environmental impact, aligning with global sustainability goals while maintaining efficient metal recovery. Furthermore, the development of smart solvent systems capable of adapting to varying extraction conditions or optimizing selectivity through programmable responses offers a promising avenue for enhancing the efficiency and precision of SX technologies.

#### 6.2.2. Integration With Other Technologies

Integrating SX with other innovative technologies can enhance overall process efficiency and sustainability. Hybrid processes that integrate SX with methods like bioleaching or electrochemical techniques can leverage the strengths of each approach, improving metal recovery rates by enhancing pretreatment and subsequent extraction steps. Additionally, advanced separation techniques, such as membrane filtration or adsorption, can complement SX by enabling selective metal separation before or after the extraction process, thereby boosting overall recovery efficiency and process performance.

#### 6.2.3. Policy Support and Industry Collaboration

The successful implementation of SX technologies within the circular economy can be facilitated through supportive policies and collaborative efforts between stakeholders. Government initiatives, such as financial support, tax incentives, and grants for research and development, can play a crucial role in promoting the adoption of sustainable technologies like SX, encouraging industries to invest in innovative solutions that align with sustainability goals. Additionally, partnerships between academia, industry, and research institutions can drive progress in SX technologies by fostering knowledge sharing, accelerating the development of new methods, and enhancing the scalability of advanced processes.

#### 6.2.4. Long‐Term Implication for Industrial Sustainability

The integration of SX into circular economy frameworks carries profound implications for the long‐term sustainability of industrial sectors, particularly those dependent on critical metals. By enabling the recovery and reuse of scarce resources, SX reduces the pressure on primary mining, mitigates the environmental impacts of metal extraction, and contributes to the conservation of natural ecosystems. Moreover, as industries increasingly adopt circular economy principles, SX will likely become a cornerstone of sustainable manufacturing processes. Its flexibility in handling diverse waste streams and potential to recover high‐value materials position it as an essential tool in driving the transition toward sustainable industrial systems. Despite the advantages of SX, several challenges limit its large‐scale implementation in circular economy systems. Solvent degradation during repeated extraction cycles can reduce process efficiency and increase operational cost. In addition, certain organic solvents may pose environmental and toxicity concerns if not properly managed. Economic feasibility also remains a significant challenge, particularly for advanced solvent systems such as ILs, which often involve high synthesis and regeneration costs. These factors highlight the need for more sustainable, cost‐effective, and recyclable solvent systems for industrial applications. Thus, by addressing the challenges associated with SX while capitalizing on opportunities for innovation is essential for enhancing the role of this technology in the circular economy. By developing greener solvents, optimizing processes, and fostering collaborative efforts, SX can evolve into a more effective and sustainable solution for metal recovery, thereby contributing to resource conservation and environmental sustainability in the years to come.

## 7. Critical Questions and Future Research Directions

Although SX technologies have demonstrated considerable promise in metal recovery and recycling, several critical questions remain insufficiently addressed. These knowledge gaps must be tackled to fully realize the environmental and economic benefits of SX systems.

### 7.1. Solvent Loss and Stability

One of the most pressing concerns is the extent and variability of solvent loss during operation. Different solvent systems, including ILs, DES, and traditional organic solvents, differ markedly in volatility, chemical stability, and resistance to degradation. However, systematic comparative studies quantifying solvent loss under continuous operation are limited. Understanding these losses is essential to assess environmental emissions, health risks, and overall process sustainability.

### 7.2. Cost‐Effectiveness at Scale

Though many solvent systems show promising extraction efficiencies at laboratory or pilot scale, the economic feasibility of scaling up remains uncertain. Large‐scale deployment introduces complexities such as solvent regeneration costs, process integration, and handling of by‐products. Detailed techno‐economic analyses are needed to evaluate the cost‐benefit balance of different solvents and processes in industrial settings, considering capital expenditures and operational expenditures holistically.

### 7.3. Energy Payback and Life Cycle Impact

Energy consumption is a critical factor in determining the sustainability of SX technologies. The energy payback time, defined as the duration required for the system to recover the energy invested in solvent production, operation, and recovery, is rarely reported. Quantifying this parameter through LCA methodologies would provide insights into the net environmental impacts and guide the optimization of energy efficiency in closed‐loop SX systems.

Addressing these questions through rigorous experimental studies and modeling will be pivotal for advancing SX technologies from promising laboratory concepts to robust, sustainable industrial practices. Future research should emphasize cross‐comparisons of solvent systems under standardized conditions, integrate techno‐economic and environmental assessments, and develop strategies to minimize solvent losses and energy demands.

## 8. Future Perspective

Although the current applications of SX are well established in metal recovery and waste valorization, emerging literature suggests exciting opportunities in the field of carbon capture and CO_2_ mineralization. Advanced SX systems—utilizing task‐specific ILs or CO_2_‐philic DES—have demonstrated early potential for selectively extracting CO_2_ or facilitating carbonation reactions. Additionally, integrating SX into processes that extract Mg^2+^ or Ca^2+^ from industrial residues could support efficient mineral carbonation and carbon‐negative material production. These interdisciplinary opportunities remain largely untapped and warrant further research, particularly in terms of solvent system design, life cycle performance, and process integration with existing CO_2_ utilization or sequestration frameworks. Exploring such novel directions could significantly expand the role of SX in addressing global climate and sustainability challenges.

### 8.1. Potential Role of SX in Carbon Capture and CO_2_ Mineralization

As the world strives toward climate neutrality, carbon capture and CO_2_ mineralization have gained significant attention. SX, traditionally employed in hydrometallurgy, is now emerging as a promising tool in these domains due to its selectivity and compatibility with advanced green solvents.

#### 8.1.1. SX in CO_2_ Capture

Green solvents like ILs and DES have shown significant promise in postcombustion CO_2_ capture due to their high affinity for CO_2_, thermal stability, and reusability. These solvents are increasingly used in SX systems, enabling the selective separation of CO_2_ from industrial flue gases. Coupling SX with ILs/DES–based systems can improve energy efficiency by reducing solvent loss and regeneration costs.

#### 8.1.2. SX in CO_2_ Mineralization

Mineral carbonation involves reacting CO_2_ with metal ions (e.g., Ca^2+^ and Mg^2+^) to form stable carbonates. SX can facilitate this process by extracting these metals from ores, industrial slags, or brines. Recent studies suggest that IL‐based SX systems can effectively concentrate and purify these ions, enhancing the kinetics of carbonate formation while enabling the simultaneous recovery of valuable materials.

#### 8.1.3. Outlook and Research Opportunities

The integration of SX in carbon management opens avenues for scalable, sustainable CO_2_ mitigation strategies. Future research should focus on developing tailored extractants, optimizing process conditions, and exploring industrial symbiosis between metal recovery and carbon sequestration.

### 8.2. Integration of Digital Technologies: Machine Learning (ML) and Digital Twins in SX

Recent developments in digital technologies are poised to revolutionize SX processes by enabling smarter, data‐driven, and more adaptive systems. Among these, ML and digital twins are emerging as powerful tools for optimization, control, and sustainability assessment.

#### 8.2.1. ML for Process Optimization

ML algorithms such as supervised, unsupervised, and reinforcement learning are increasingly applied to predict and optimize critical parameters like solvent composition, temperature, pH, and flow rate. By processing large datasets from experimental or industrial sources, these models can uncover hidden patterns and recommend optimal conditions for maximum recovery and minimal environmental impact. For *example*, a recent study utilized ML techniques to predict solvent recovery efficiencies and optimize extraction parameters for the recovery of REEs from waste electronics, resulting in energy savings and enhanced resource utilization.

#### 8.2.2. Digital Twins for Real‐Time Monitoring and Control

Digital twins are virtual representations of physical SX systems that integrate real‐time sensor data with mechanistic and data‐driven models. These dynamic systems allow simulation of various operating scenarios, prediction of process behavior under disturbances, and implementation of predictive maintenance strategies. For *example*, in the mining industry, digital twin models have been used to enhance hydrometallurgical processes, and similar approaches are now being explored for SX systems to improve energy efficiency, reduce downtime, and monitor solvent degradation. Artificial intelligence and digitalization can support SX processes through predictive modeling, optimization of extraction conditions, and real‐time monitoring of system performance. ML approaches may assist in solvent selection, process control, and reduction of chemical waste, thereby improving the efficiency and sustainability of SX within circular economy frameworks.

These technologies are aligned with the principles of green chemistry and circular economy by promoting resource efficiency, real‐time adaptability, and low‐waste operations. Their broader implementation in SX will likely play a transformative role in future sustainable process engineering.

### 8.3. Research Priorities and Roadmap for the Next Decade

To further advance SX as a cornerstone of the circular economy, several key research and innovation gaps must be addressed over the next 5–10 years. These include the following:

#### 8.3.1. Solvent Design and Innovation

Although ILs, DES, and biobased extractants show promise, challenges remain in their cost, biodegradability, selectivity in complex mixtures, and large‐scale applicability. Future funding should prioritize

▪ Task‐specific green solvent development with tunable selectivity,
▪ Cheminformatics and AI tools for rapid solvent screening, and
▪ Recyclability and low‐toxicity solvent systems aligned with green chemistry principles.

#### 8.3.2. Advanced Reactor and Process Engineering

Conventional mixer‐settler systems are often inefficient in terms of space, energy, and scalability. Priority areas include

▪ Intensified reactors (e.g., microfluidic and membrane‐assisted SX units),
▪ Modular, portable SX systems for decentralized recycling (e.g., batteries and e‐waste), and
▪ Integration of sensors for real‐time feedback and process control.

#### 8.3.3. Integration With Complementary Technologies

To achieve closed‐loop recovery systems, SX must be more effectively coupled with

▪ Ion exchange, precipitation, electrochemical methods, and membrane processes,
▪ Hybrid flowsheets for resource recovery and CO_2_ valorization, and
▪ Industrial symbiosis to utilize by‐products and waste streams.

#### 8.3.4. Digitalization and AI Integration

The adoption of digital twins, ML, and automation can dramatically increase process efficiency:

▪ AI‐driven decision‐making for dynamic SX control,
▪ Predictive maintenance and process optimization, and
▪ Real‐time adaptability to changes in feedstock composition

#### 8.3.5. Policy, Funding, and Interdisciplinary Collaboration

Finally, cross‐sector efforts are essential to support SX innovation:

▪ Funding for pilot‐scale projects and industrial demonstrations,
▪ Policies incentivizing green separation technologies, and
▪ Collaboration between chemists, engineers, and data scientists to design integrated, circular systems.

**Figure 1:**
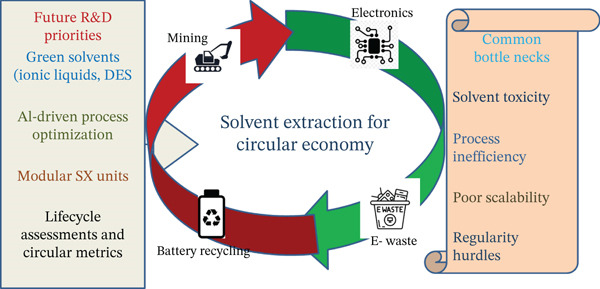
Roadmap illustrating the role of solvent extraction (SX) across various circular economy domains, including primary mining, electronics recycling, and battery recovery. Figure 1 highlights current bottlenecks and outlines priority areas for future research and development to promote sustainable and efficient SX processes.

To provide a clear overview of the multifaceted role of SX within the circular economy, Figure 1 presents a roadmap that summarizes key application domains—including mining, e‐waste recycling, and battery recovery. Figure 1 also identifies current bottlenecks such as solvent toxicity and process inefficiencies, and highlights future research priorities including green solvent development, process intensification, and digital integration. This visual roadmap underscores the critical challenges and opportunities that must be addressed to advance sustainable SX technologies.

## 9. Limitations and Variability of Reported Data

The quantitative values discussed in this review are derived from a combination of laboratory‐scale, pilot‐scale, and limited industrial‐scale studies reported in the literature. Reported recovery efficiencies and environmental performance indicators vary significantly depending on factors such as solvent composition, metal concentration, feed characteristics, operating conditions, and process scale. For example, several studies reporting extraction efficiencies above 90% were conducted under optimized laboratory conditions, which may not fully reflect industrial operational environments. Furthermore, direct comparison among studies remains challenging due to differences in experimental methodologies and evaluation criteria. Therefore, the reported performance metrics should be interpreted cautiously, and additional large‐scale validation studies are necessary to assess the long‐term industrial feasibility and sustainability of SX technologies within circular economy applications. Despite significant progress in SX technologies, several research gaps remain unresolved. Limited industrial‐scale validation, insufficient long‐term environmental assessment, solvent recyclability challenges, and the absence of standardized sustainability evaluation metrics continue to restrict broader industrial implementation. Furthermore, additional research is required to improve the integration of green solvent systems, LCA approaches, and AI‐driven process optimization tools for more efficient and sustainable circular economy applications.

## 10. Conclusion

SX has emerged as a pivotal technology in advancing the principles of the circular economy, offering a sustainable pathway for the recovery of valuable metals from diverse waste streams. This review has underscored its multifaceted advantages, including enhanced resource efficiency, reduction in waste generation, and mitigation of GHG emissions. By enabling the transformation of industrial residues and end‐of‐life products into secondary resources, SX contributes significantly to environmental sustainability and economic resilience. Despite its proven efficacy, the widespread adoption of SX is constrained by several critical challenges. These include the need for environmentally benign and cost‐effective solvents, optimization of process parameters for large‐scale applications, and competition from alternative technologies. Addressing these challenges demands continued innovation, particularly in the development of green solvents, hybrid systems, and process intensification strategies. Recent progress in the integration of SX with complementary techniques, such as biohydrometallurgy and digital process control, signals a promising trajectory for the field. Moreover, the alignment of SX with global sustainability goals and policy frameworks further strengthens its relevance in modern industrial practices. In conclusion, the future of SX within circular economy models is promising. With sustained research efforts, cross‐sectoral collaboration, and supportive policy environments, SX can play a transformative role in closing material loops, reducing environmental footprints, and fostering a more sustainable and economically viable industrial ecosystem.

## Funding

No funding was received for this manuscript.

## Conflicts of Interest

The author declares no conflict of interest.

## Data Availability

Data sharing is not applicable to this article as no datasets were generated or analyzed during the current study.
